# Recombination Activating Gene-2 Regulates CpG-Mediated Interferon-α Production in Mouse Bone Marrow-Derived Plasmacytoid Dendritic Cells

**DOI:** 10.1371/journal.pone.0047952

**Published:** 2012-10-24

**Authors:** Xin M. Luo, Margarida Y. Y. Lei

**Affiliations:** 1 Department of Biomedical Sciences and Pathobiology, Virginia-Maryland Regional College of Veterinary Medicine, Virginia Polytechnic Institute and State University, Blacksburg, Virginia, United States of America; 2 Division of Biology, California Institute of Technology, Pasadena, California, United States of America; Kantonal Hospital St. Gallen, Switzerland

## Abstract

Using mice that lack recombination activating gene-2 (Rag2), we have found that bone marrow-derived plasmacytoid dendritic cells (pDCs) as main producers of interferon-α (IFNα) require Rag2 for normal development. This is a novel function for Rag2, whose classical role is to initiate B and T cell development. Here we showed that a population of common progenitor cells in the mouse bone marrow possessed the potential to become either B cells or pDCs upon appropriate stimulations, and the lack of Rag2 hindered the development of both types of progeny cells. A closer look at pDCs revealed that Rag2^−/−^ pDCs expressed a high level of Ly6C and were defective at producing IFNα in response to CpG, a ligand for toll-like receptor 9. This phenotype was not shared by Rag1^−/−^ pDCs. The induction of CCR7, CD40 and CD86 with CpG, however, was normal in Rag2^−/−^ pDCs. In addition, Rag2^−/−^ pDCs retained the function to promote antibody class switching and plasma cell formation through producing IL-6. Further analysis showed that interferon regulatory factor-8, a transcription factor important for both IFNα induction and pDC development, was dysregulated in pDCs lacking Rag2. These results indicate that the generation of interferon response in pDCs requires Rag2 and suggest the lymphoid origin of bone marrow-derived pDCs.

## Introduction

Plasmacytoid dendritic cells (pDCs) were first described as “plasma cell-like” by pathologists [1] and later found to be identical to the “professional interferon-producing cells” in the peripheral blood and secondary lymph organs [2], [3]. Their primary function is to produce type I interferons but they also produce other cytokines and are involved in antigen presentation [4], [5]. They are considered a functional link between innate and adaptive immune responses.

Whether pDCs originate from the myeloid or lymphoid lineage has been a focus of debate for the last decade. The myeloid hypothesis is supported by the observations that Flt3^+^ myeloid progenitors can generate pDCs [6] and that the depletion of common lymphoid progenitors does not appear to affect pDC generation [7]. However, more evidence supports the lymphoid hypothesis. First, adoptive transfer experiments have shown the generation of pDCs from lymphoid progenitors [6]. Second, common myeloid progenitor-derived pDCs have been shown to express lymphoid-associated genes such as pre-Tα and recombination activating gene-1 (Rag1) [8]. Third, pDCs share many molecular features of B cells, such as the expression of B220, Tdt, VpreB, Rag1, Rag2, and D-to-J rearrangement of the immunoglobulin (Ig) heavy chain locus [8]–[10]. Why pDCs need to express B cell-specific genes and rearrange the Ig heavy chains locus is unknown but it raises the possibility that pDCs and B cells may have differentiated from the same progenitor cells.

Rag proteins play an essential role in V(D)J recombination by inducing site-specific cleavage and recombination of variable (V), joining (J), and sometimes diversity (D) gene segments that are initially separated in the germline configuration [11]–[13]. Although they were originally identified in T and B cells [14], [15], Rag1 and Rag2 transcripts have been found in non-T/B cells such as dendritic cells [8], [16] and natural killer cells [17]. In addition, Rag1 has been shown to function in neurogenesis in the central nervous system [18], [19]. However, whether and how Rag proteins may play a role in non-T/B cells remain unclear.

Here we show that Rag2 is required for toll-like receptor 9 (TLR9)-mediated induction of IFNα in bone marrow-derived pDCs. Our results show that, although the numbers and expansion of pDCs are similar between wildtype and Rag2^−/−^ mice, Rag2^−/−^ pDCs appear to be defective at producing IFNα in vitro and in vivo in response to the TLR9 ligand, CpG. In contrast, Rag2^−/−^ pDCs retain the functions to (1) upregulate functional surface markers CCR7, CD40 and CD86 in response to CpG and (2) promote antibody class switching and plasma cell formation, indicating that Rag2 specifically regulates CpG-induced IFNα production. In addition, we show that interferon regulatory factor-8 (IRF8), an essential transcription factor that regulates pDC development, is dysregulated in pDCs lacking Rag2. These results suggest Rag2 as an important regulator of interferon response in pDCs.

## Materials and Methods

### Ethics Statement

This study was carried out in strict accordance with the recommendations in the Guide for the Care and Use of Laboratory Animals of the National Institutes of Health. Rag1^−/−^ and Rag2^−/−^ mice, which were of BALB/c background, had been housed at California Institute of Technology (Animal Assurance Number: A3426-01) for several years prior to the start of the study. Age-matched wildtype mice were purchased from Jackson Laboratory. The protocol was approved by the Institutional Animal Care and Use Committee (IACUC) of California Institute of Technology (IACUC protocol number 1403-05T). All efforts were made to minimize suffering and mice were euthanized by CO_2_ inhalation followed by exsanguination by transcardiac blood collection.

### Cell Isolation and Culture

For the isolation of progenitor cells, bone marrow cells were sorted using a FACSAria sorter (BD Biosciences) for a population that was lineage**^−^**B220^+^Flt3^+^CD43^+^CD24^+^, where the lineage markers were Ly6C, PDCA-1, CD11c, Ter-119, and CD3e. For other experiments, bone marrow cells and splenocytes were isolated and sorted for different cell populations using magnetic-activated cell separation (MACS) kits (Miltenyi Biotec). The isolation steps were repeated whenever necessary to achieve 90% purity as determined by flow cytometry. Mouse CD19 microbeads were used for the isolation of B cells. Mouse PDCA-1 microbeads were used for the isolation of pDCs. Ly6C^hi^ and Ly6C^−/low^ cells were also separated with MACS. Cells were cultured in RPMI 1640 medium supplemented with 10% fetal bovine serum, 2 mM L-glutamine, 50 µM β-mercaptoethanol, 10 mM HEPES, 1 mM sodium pyruvate, 1× MEM nonessential amino acids, and penicillin/streptomycin. In some experiments, cells were cultured with 100 ng/mL mouse Flt3 ligand (R & D Systems), 5 µM of mouse Type A CpG oligonucleotide ODN1585 (InvivoGen), 10 ng/mL mouse IL-7 (R & D Systems), or 0.5 µg/mL of an anti-mouse IL-6 blocking antibody or isotype control (eBioscience).

### Reverse Transcription-quantitative PCR (RT-qPCR)

Total RNA was isolated from total bone marrow cells or MACS-sorted cells (>90% purity as determined by flow cytometry) using RNeasy Mini Kit (Qiagen) per manufacturer’s instructions. Reverse transcription was performed by using iScript™ cDNA Synthesis Kit (Bio-Rad). SYBR Green-based quantitative real-time PCR was conducted with a 7300 Realtime PCR system (Applied Biosystems) to assay Rag1, Rag2, IRF8, and L32 mRNA amounts with gene-specific primers (sequences available upon request). For all experiments, mRNA expression was normalized to that of a relatively stable ribosomal protein L32 [20].

### Wright’s Stain

For Wright’s stain, single cell suspensions were cytospinned onto slides, air-dried, stained, and examined on an Olympus BX-51 microscope and photographed using a Spot Digital Camera.

### Flow Cytometry

Fluorophore-conjugated monoclonal antibodies specific to PDCA-1, B220, Ly6C, CD11c, CD11b, TLR9 (intracellular), CD40, CD86, CCR7, or CD138 (eBioscience) were used to stain cells. After washing, stained cells were assayed with a BD FACSCalibur flow cytometer (BD Biosciences). Results were processed with FlowJo software (Tree Star).

### Enzyme-linked Immunosorbent Assays (ELISAs)

Mouse IFNα, IL-6, IgM, and IgG2a ELISAs were performed with cytokine-specific kits (PBL Interferon Source or eBioscience) or antibody-specific kits (Bethyl Laboratories) and carried out according to the manufacturer’s instructions.

### In vivo CpG Stimulation

To prepare each milliliter of the ODN1585/1,2-dioleoyl-3-trimethylammonium-propane (DOTAP) complex for in vivo CpG stimulation, 100 µL of 500 µM ODN1585 was mixed with 20 µL of PBS and 30 µL (30 µg) of DOTAP (Roche), incubated for 15 min at room temperature, and then diluted with 850 µL of PBS to make 50 µM of the complex. For each mouse, 150 µL of ODN1585/DOTAP complex was injected intravenously.

### Statistical Analysis

Student’s two-tailed *t* tests were performed using Microsoft Excel statistical software module (Microsoft). All data, unless specified, are shown as the mean + standard error, and the difference was considered statistically significant when the *P* value was less than.05.

## Results

### Requirement for Rag2 in pDC Development

**Figure 1:**
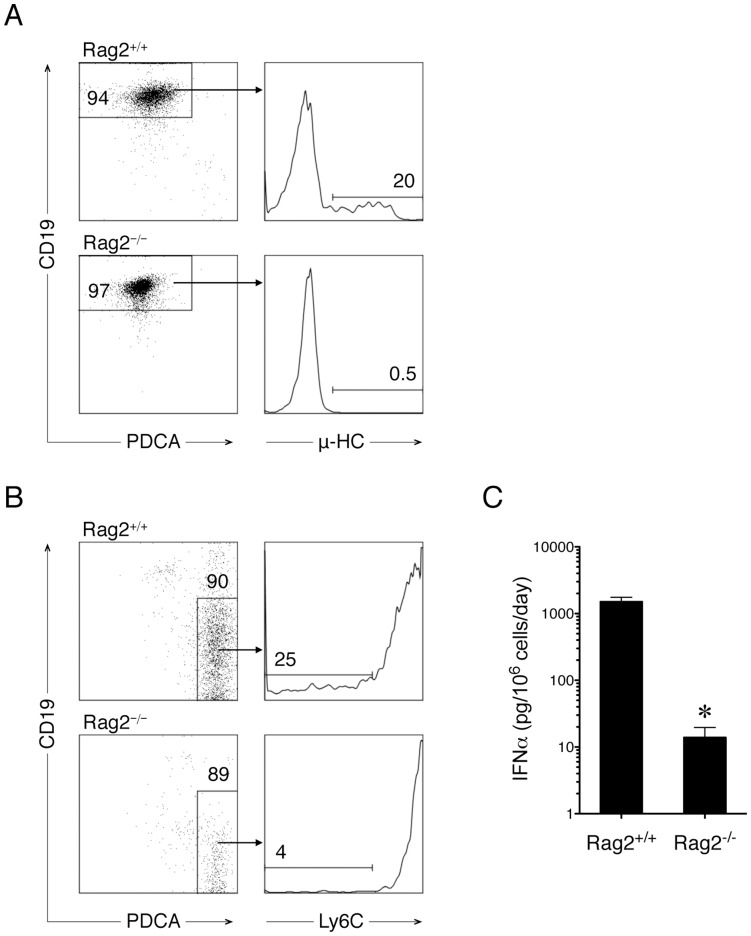
Generation of B cells and pDCs from a common population of mouse progenitor cells. Bone marrow cells were isolated from Rag2^+/+^ and Rag2^−/−^ mice, sorted with a FACSAria sorter for a population that was lineage**^−^**B220^+^Flt3^+^CD43^+^CD24^+^, where the lineage markers were Ly6C, PDCA-1, CD11c, Ter-119, and CD3e. *A.* Generation of B cells from the sorted cells. The sorted cells (2 × 10^5^ sorted cells/200 µL/well) were cultured in the presence of IL-7 for 4 days and analyzed by using flow cytometry. Over 90% of the derived cells were CD19^+^ and some of them also expressed surface Igμ heavy chain (μ-HC), indicating the presence of pre-B cells. *B*. Generation of pDCs from the sorted cells. The sorted cells (2 × 10^5^ sorted cells/200 µL/well) were cultured in the presence of Flt3 ligand and ODN1585 for 4 days and analyzed by flow cytometry. About 90% of progeny cells were PDCA-1^+^. The expression levels of Ly6C on the surface of PDCA-1^+^ cells are also shown. Representative plots of 3 independent experiments are shown. *C*. IFNα production from the generated pDCs. The unit of measurement for IFNα production, pg/10^6^ cells/day, was based on the starting number of sorted cells when they were seeded. The difference in IFNα production was significant (n  = 3, *p*<.05).

In the mouse bone marrow, Rag1 and Rag2 proteins are expressed in early stages of B cells to regulate V(D)J rearrangement of the Ig heavy and light chain loci. PDCs have also been shown to express Rag proteins and undergo D-to-J rearrangement [9], raising the possibility that pDCs and B cells may have differentiated from the same progenitor cells. Thus, we sorted Hardy fractions B-C from mouse bone marrow cells and examined the potential of the sorted cells to generate B cells and pDCs, respectively. These fractions of cells were previously defined as pre-pro-B and pro-B cells (lineage**^−^**B220^+^Flt3^+^CD43^+^CD24^+^) and had D-to-J, but not V-to-DJ rearrangements [21]. As anticipated, the sorted progenitor cells generated CD19^+^μ^+^ pre-B cells in vitro upon stimulation with mouse IL-7 (Fig. 1*A*, upper panels) and the expression of μ heavy chain was abolished in the absence of Rag2 (Fig. 1*A*, lower panels). Importantly, the same progenitor cells were also able to generate IFNα-producing PDCA-1^+^ pDCs in the presence of Flt3 ligand and a TLR9 ligand (Type A CpG oligonucleotide ODN1585), regardless of Rag2 expression (Fig. 1*B–C*). However, we observed that pDCs generated from Rag2^−/−^ progenitors expressed a higher level of Ly6C (Fig. 1*B*) and produced significantly less IFNα than Rag2^+/+^ pDCs (Fig. 1*C*; *p*<.05). These results indicate that B cells and pDCs may be generated from a common population of progenitor cells and that Rag2 may be required for the development of both types of progeny cells.

**Figure 2:**
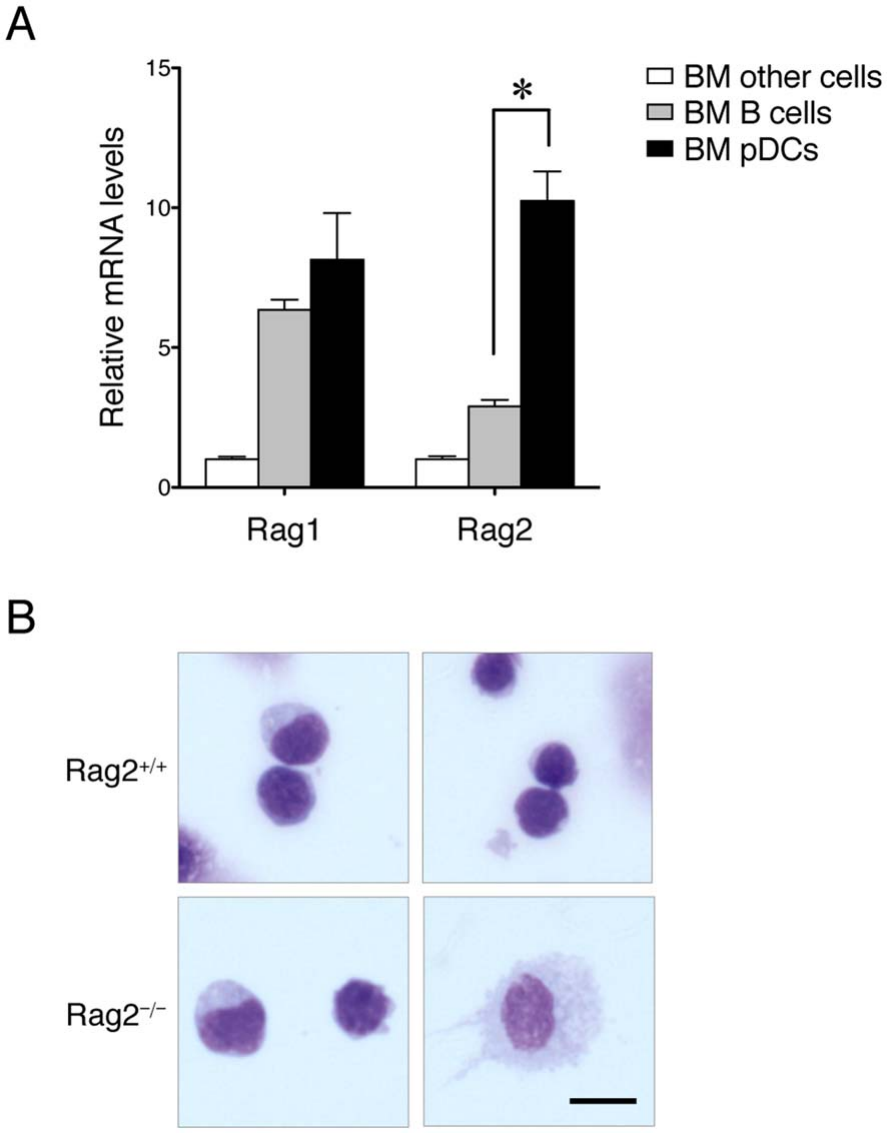
Preferential expression of Rag2 in pDCs. *A.* Rag1 and Rag2 expression in B cells, pDCs, and other cells in the mouse bone marrow. Bone marrow (BM) cells were isolated from wildtype mice and sorted for B cell, pDCs, and other cells. Total RNA was isolated from different subpopulations of cells and RT-qPCR was performed. Rag1 and Rag2 mRNA levels were normalized to L32 and the expression levels in non-B/non-pDC cells were defined as 1. The difference in Rag2 expression between B cells and pDCs was significant (n  = 3, *p*<.05). *B.* Morphologic difference between bone marrow-derived Rag2^+/+^ and Rag2^−/−^ pDCs. Bone marrow cells were isolated from Rag2^+/+^ and Rag2^−/−^ mice and pDCs were sorted. Single cell suspensions were cytospinned onto slides, air-dried, stained, and examined on an Olympus BX-51 microscope (40× objective lens) and photographed using a Spot Digital Camera. Two representative images for each type of mice are shown. Bar represents 5 µm.

We next compared the expression of Rag1 and Rag2 in pDCs versus B cells and other cells in the bone marrow. As anticipated, both Rag1 and Rag2 were highly expressed in pDCs and B cells but not in other cells (Fig. 2*A*). Importantly, we found that while Rag1 was expressed equally in pDCs and B cells, the expression of Rag2 was 3-fold higher in pDCs than in B cells (*p*<.05), indicating that Rag2 is preferentially expressed in pDCs. This indicates that Rag2 might have a unique function in pDCs. In addition, we found that bone marrow-derived Rag2^−/−^ pDCs were morphologically different from Rag2^+/+^ pDCs (Fig. 2*B*). While Rag2^+/+^ pDCs were uniformly “plasma cell-like” under the microscope, there appeared to be two subpopulations of Rag2^−/−^ PDCA-1^+^ pDCs in the bone marrow–one was morphologically similar to Rag2^+/+^ pDC but the other possessed the morphology of activated interdigitating pDCs [1]. This also suggests that Rag2 may have an unknown function in pDCs.

### Phenotypic Difference between Rag2^+/+^ and Rag2^−/−^ pDCs

**Figure 3:**
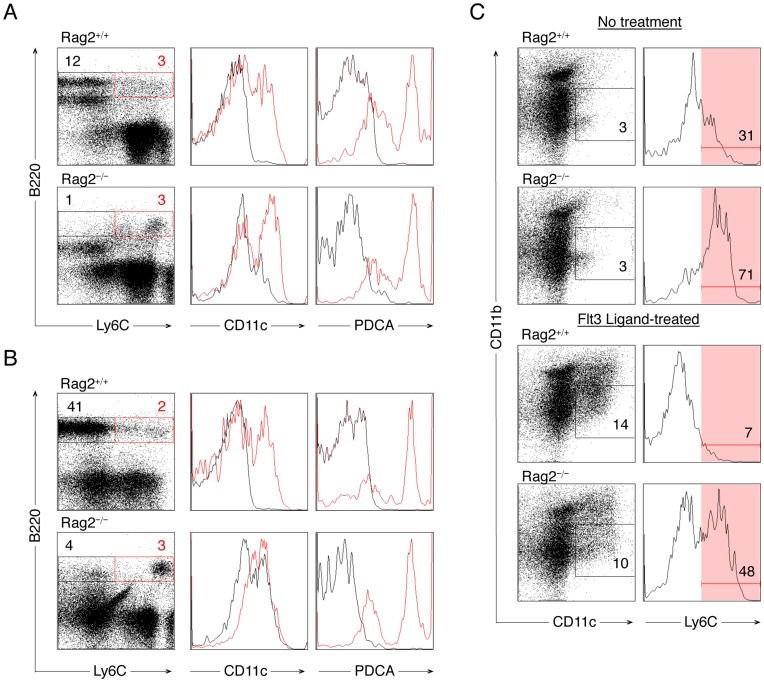
Rag2^+/+^ and Rag2^−/−^ pDCs different in Ly6C expression. *A.* Analysis of bone marrow cells isolated from Rag2^+/+^ and Rag2^−/−^ mice. B220^hi^Ly6C**^−^** cells were gated with a black gate and are shown as black lines in the CD11c and PDCA-1 histograms. B220^hi^Ly6C^+/low^ cells were gated with a red gate and are shown as red lines in the CD11c and PDCA-1 histograms. Representative plots of 3 independent experiments are shown. *B.* Analysis of splenocytes isolated from Rag2^+/+^ and Rag2^−/−^ mice. B220^+^Ly6C**^−^** and B220^+^Ly6C^+/low^ cells were gated and analyzed as described in *A*. Note that in both bone marrow and spleen, the expression level of Ly6C in the Rag2^−/−^ red gate was higher than that in the Rag2^+/+^ red gate. *C.* Ly6C expression during Flt3 ligand-mediated expansion of CD11b**^−^**CD11c^+^ cells. Total bone marrow cells were either untreated or treated with Flt3 ligand for 5 days. CD11b**^−^**CD11c^+^ cells were gated and analyzed for Ly6C expression. Representative plots of 3 independent experiments are shown.

PDCs are phenotypically defined as B220^+^CD11c^+/low^PDCA-1^+^ cells in mice [1]. Siglec-H, another pDC-specific marker, is uniformly expressed on B220^+^CD11c^+/low^PDCA-1^+^ cells that have not been previously stimulated [22]. Although PDCA-1 is expressed at a low level in some subpopulations of B cells and inducible in many cells upon activation [23], we chose to use a relatively high level of PDCA-1 as one specific marker for pDCs in the analysis and sorting of unstimulated cells because antibodies against Siglec-H have been shown to inhibit IFNα production in pDCs [24]. PDCs also react with antibodies that recognize Ly6C but it is controversial whether Ly6C^hi^ or Ly6C^low^ pDCs, or both, are capable of producing IFNα [16], [25]–[27]. We found that bone marrow B220^hi^Ly6C^+/low^ cells (shown in red gate), but not B220^hi^Ly6C**^−^** cells (shown in black gate) or B220^low^ cells (gate not shown), were positive for PDCA-1 (Fig. 3*A*). We also found that although the numbers of pDCs were similar between Rag2^+/+^ and Rag2^−/−^ mice (Fig. S1), Rag2^−/−^ pDCs expressed a higher level of Ly6C than Rag2^+/+^ pDCs (Fig. 3*A and 3B*, comparing the red gates), with the phenomenon especially evident in the spleen (Fig. 3*B*), suggesting phenotypic variation of pDCs according to Rag2 expression.

Because Ly6C is also expressed on CD11b^+^ monocytes/macrophages [28], we excluded CD11b^+^ cells by gating on CD11c^+^CD11b**^−^** cells (Fig. 3*C*). These cells were expanded by Flt3 ligand, a cytokine known to promote pDC development and proliferation [29]–[31], regardless of Rag2 expression (Fig. 3*C*). However, under both untreated and Flt3 ligand-treated conditions, Ly6C expression was higher in CD11c^+^CD11b**^−^** cells lacking Rag2 than their wildtype or Rag1^−/−^ counterparts (Fig. 3*C* and Fig. S2). These results suggest that Rag2 may be important for certain function(s) of pDCs that are related to Ly6C expression, such as the production of IFNα [16], [26], [27].

### Rag2^−/−^ pDC Defective at IFNα Production upon TLR9 Stimulation

**Figure 4:**
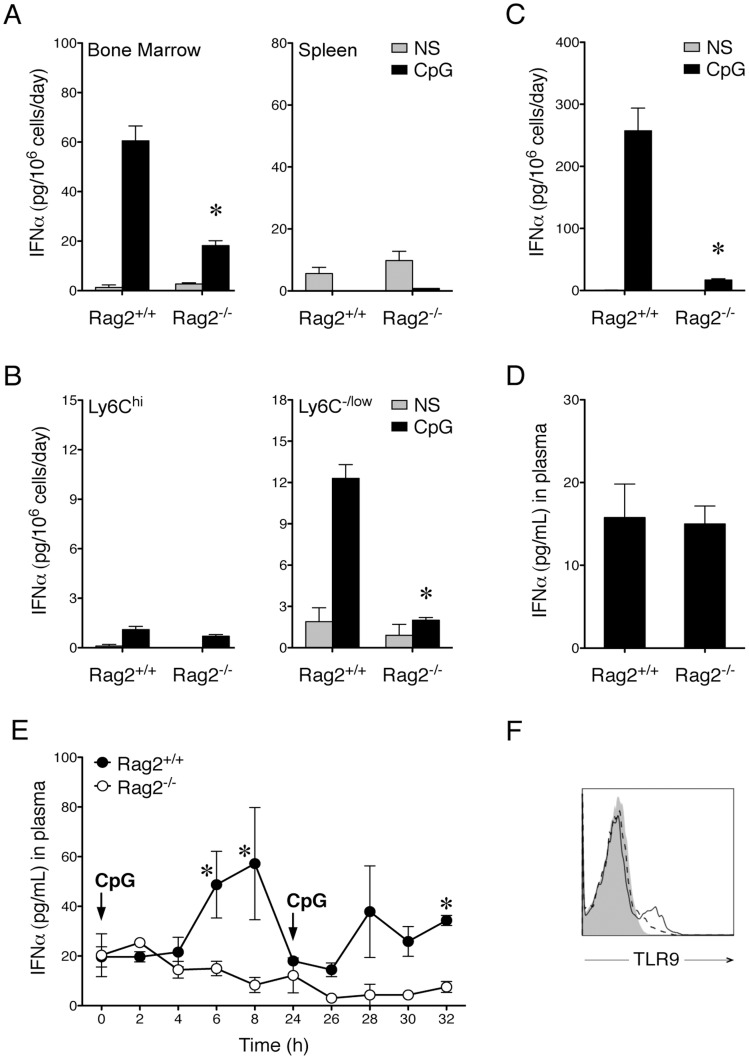
Rag2^−/−^ pDCs defective at IFNα production upon CpG stimulation. *A.* CpG-induced IFNα production in bone marrow cells but not in splenocytes. Bone marrow cells (left panel) and splenocytes (right panel) were isolated from Rag2^+/+^ and Rag2^−/−^ mice. Cells were either not stimulated (NS) or stimulated with ODN1585 (CpG) in vitro for 5 days. Production of IFNα in the medium was measured by using ELISA. The asterisk represents significant difference (n  = 4, *p*<.05) between the CpG-induced IFNα levels in Rag2^+/+^ versus Rag2^−/−^ bone marrow cells. *B.* CpG-induced IFNα production in Ly6C^−/low^ bone marrow cells. Bone marrow cells were isolated from Rag2^+/+^ and Rag2^−/−^ mice and separated according to Ly6C expression. The left panel shows the Ly6C^hi^ population whereas the right panel shows the Ly6C^−/low^ population. Cells were either not stimulated (NS) or stimulated with ODN1585 (CpG) in vitro for 2 days. Production of IFNα in the medium was measured by using ELISA. The asterisk shows that, within the Ly6C**^−^** ^/low^ population, there was significant difference (n  = 4, *p*<.05) between the CpG-induced IFNα levels in Rag2^+/+^ versus Rag2^−/−^ cells. *C.* Rag2^−/−^ PDCA-1^+^ pDCs defective at CpG-induced IFNα production. Bone marrow cells were isolated from Rag2^+/+^ and Rag2^−/−^ mice and PDCA-1^+^ pDCs were sorted. Cells were either not stimulated (NS) or stimulated with ODN1585 (CpG) in vitro for 5 days. Production of IFNα in the medium was measured and the asterisk represents significant difference (n  = 3, *p*<.05) between the CpG-induced IFNα levels in Rag2^+/+^ versus Rag2^−/−^ pDCs. *D.* Plasma levels of IFNα in Rag2^+/+^ and Rag2^−/−^ mice. The two groups are not significantly different (n  = 8; *p*>.05). *E.* In vivo response to CpG. Rag2^+/+^ and Rag2^−/−^ mice (n  = 3 per group) were treated with ODN1585/DOTAP complex (CpG) intravenously at 0 and 24 h. Mice were tail-bled every 2 h for up to 8 h after each CpG injection. Plasma levels of IFNα were measured by using ELISA. The asterisks represent significant differences (*p*<.05) between plasma IFNα levels in Rag2^+/+^ versus Rag2^−/−^ mice at 6, 8, and 32 h. *F*. TLR9 expression. Bone marrow cells were isolated from Rag2^+/+^ and Rag2^−/−^ mice and stained for surface PDCA-1 and intracellular TLR9 expression. The histogram shown is a representation of 3 independent experiments and has been pre-gated on PDCA-1^+^ cells. The shaded peak represents isotype control and the dashed and solid lines represent Rag2^+/+^ and Rag2^−/−^ pDCs, respectively.

PDCs express and secrete IFNα in response to viral infection [32], [33] or TLR ligation [1], [34]. To determine the effect of Rag2 on this primary function of pDCs, we stimulated wildtype, Rag1^−/−^, or Rag2^−/−^ bone marrow cells with CpG in vitro. Our result showed that while wildtype (Rag2^+/+^) and Rag1^−/−^ bone marrow cells produced a large amount of IFNα in response to CpG, the induction of IFNα was significantly lower in Rag2^−/−^ bone marrow cells (Fig. 4*A*, left panel, *p*<.05; and Fig. S3), suggesting that Rag2 may be required for TLR9-mediated production of IFNα in the bone marrow. Neither Rag2^+/+^ nor Rag2^−/−^ cells derived from the spleen produced a significant level of IFNα upon CpG stimulation (Fig. 4*A*, right panel), consistent with previous reports [35], [36]. Therefore, we focused on bone marrow cells in the following experiments.

Different subpopulations of pDCs in the mouse bone marrow have been found with differential IFNα-producing capacity [16], [25]–[27], [36]. With regard to Ly6C expression, both Ly6C^hi^ pDCs [16], [25] and Ly6C^low^ pDCs [26], [27] have been shown to produce IFNα. We found that isolated bone marrow cells that expressed a high level of Ly6C, which might include both pDCs and monocytes/macrophages, were poor producers of IFNα in response to CpG stimulation (Fig. 4*B*, left panel). The Ly6C^−/low^ population of Rag2^+/+^ bone marrow cells, on the other hand, responded well to CpG and produced a large amount of IFNα (Fig. 4*B*, right panel), suggesting that the production of IFNα may correlate with a lower expression level of Ly6C. However, the Ly6C^−/low^ fraction of Rag2^−/−^ bone marrow cells produced a significant lower amount of IFNα than their Rag2^+/+^ counterparts in response to CpG stimulation (Fig. 4*B*, right panel; *p*<.05), likely because there were fewer Ly6C^low^ pDCs in Rag2^−/−^ bone marrow cells (Fig. 3*A and 3C*). In addition, we found that isolated PDCA-1^+^ Rag2^−/−^ pDCs produced a significantly lower level of IFNα than isolated PDCA-1^+^ Rag2^+/+^ pDCs upon CpG stimulation (Fig. 4*C*; *p*<.05). These results suggest that Rag2^−/−^ pDCs, expressing a high level of Ly6C, are defective at producing IFNα upon TLR9 ligation in vitro. We also tested the effect of TLR7/8 ligand Gardiquimod on bone marrow-derived pDCs and did not find any difference in IFNα production with or without Rag2 (data not shown), suggesting that the defect in Rag2^−/−^ pDCs may be restricted to TLR9-mediated induction of IFNα.

We next examined the effect of Rag2 on IFNα production in vivo. The basal concentrations of IFNα in the mouse plasma were similar in Rag2^+/+^ and Rag2^−/−^ mice (Fig. 4*D*). However, the CpG-induced level of IFNα appeared to be different between the two types of mice (Fig. 4*E*). To increase CpG uptake by pDCs in vivo, we used a cationic lipid DOTAP that helps the localization of negatively charged CpG oligonucleotide in the early endosomes [37], [38] and injected the CpG/DOTAP complex intravenously to target pDCs in the peripheral blood and bone marrow. Our result showed that IFNα was rapidly induced upon CpG stimulation in Rag2^+/+^ mice (Fig. 4*E*). The IFNα-producing cells became refractory to a secondary stimulation, consistent with the reported kinetics of IFNα response in pDCs [39]. In contrast, Rag2^−/−^ mice did not have any change in IFNα levels in response to either primary or secondary CpG stimulation (Fig. 4*E*), suggesting that Rag2 may be required for TLR9-mediated IFNα production in vivo. Lastly, we confirmed that the defect of IFNα production in Rag2^−/−^ pDCs was not due to a decrease in TLR9 expression (Fig. 4*F*).

### Rag2^−/−^ pDC Normal in other Functions

**Figure 5:**
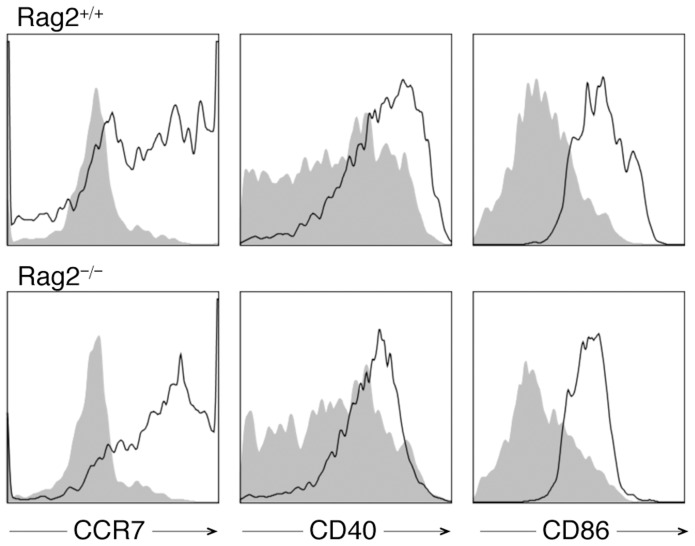
CCR7, CD40 and CD86 induction with CpG in PDCA-1^+^ cells regardless of Rag2 expression. Bone marrow cells were isolated from Rag2^+/+^ and Rag2^−/−^ mice and stimulated with ODN1585 in vitro for 4 days. The expression levels of CCR7, CD40, and CD86 in untreated (shaded peaks) and CpG-stimulated (solid lines) bone marrow cells are shown. The histograms shown are a representation of 3 independent experiments and have been pre-gated on PDCA-1^+^ cells.

TLR ligation in pDCs not only triggers IFNα production but also induces the expression of pDC functional markers, such as CCR7, CD40, and CD86 [33]. CCR7 mediates the migration of pDCs to lymph nodes [40], [41]. CD40L can activate pDCs through CD40 [42] and CD40L-activated pDCs can regulate adaptive immunity by inducing T helper 1 polarization in T cells [43], [44]. The co-stimulatory molecule CD86, on the other hand, has been shown to be upregulated in pDCs upon TLR ligation [44]. We found that all 3 functional markers were induced in bone marrow-derived PDCA-1^+^ cells by CpG stimulation regardless of Rag2 expression (Fig. 5), indicating that Rag2 may not be required for the induction of these markers.

**Figure 6:**
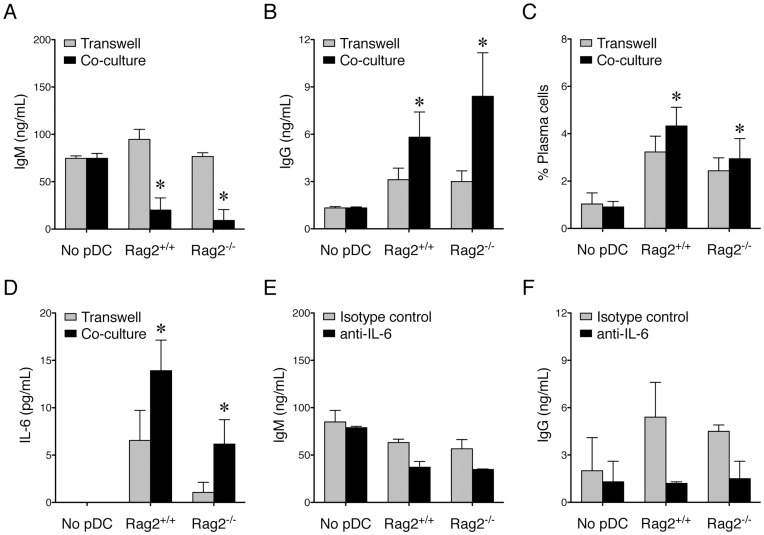
PDCs capable of supporting antibody class switching and plasma cell formation regardless of Rag2 expression. *A-C*. CD11c-depleted wildtype splenocytes (5×10^5^ cells per well) were either co-cultured, or culture in a transwell system, with medium (No pDC) or bone marrow-derived, MACS-sorted Rag2^+/+^ or Rag2^−/−^ pDCs (2.5×10^5^ sorted cells per well) for 5 days. IgM (*A*) and IgG (*B*) production was measured by using ELISAs. The percentages of CD138^+^ plasma cells at the end of the cultures are shown in *C*. The asterisks show significant differences (n  = 4, *p*<.05) compared to the No pDC control under the co-culture condition. *D.* IL-6 production by Rag2^+/+^ and Rag2^−/−^ pDCs. CD11c-depleted wildtype splenocytes were either co-cultured, or culture in a transwell system, with medium (No pDC) or sorted Rag2^+/+^ or Rag2^−/−^ pDCs for 5 days. IL-6 production was measured by using ELISA. The asterisks show significant differences (n  = 3, *p*<.05) compared to the No pDC control under the co-culture condition. *E*-*F.* Antibody class switching dependent on IL-6. CD11c-depleted wildtype splenocytes were co-cultured with medium (No pDC) or sorted Rag2^+/+^ or Rag2^−/−^ pDCs for 5 days in the presence of either isotype control or an anti-IL-6 blocking antibody. IgM (*E*) and IgG (*F*) production was measured by using ELISAs.

PDCs link innate and adaptive immunity by producing type I interferons and other cytokines [1]. One important function of pDCs in the regulation of adaptive immunity is to promote plasma cell formation and antibody class switching in B cells through producing IFNα and IL-6 [45], [46]. Because Rag2^−/−^ pDC are defective at producing IFNα (Fig. 4), we asked whether the lack of Rag2 would block the function of pDCs in antibody class switching and plasma cell formation. To examine this, CD11c-depleted wildtype splenocytes were co-cultured, or cultured in a transwell system, with bone marrow-derived Rag2^+/+^ or Rag2^−/−^ pDCs. As anticipated, Rag2^+/+^ pDCs significantly promoted antibody class switching (Fig. 6*A–B*, from IgM to IgG) and plasma cell formation (Fig. 6*C*) under the co-culture condition, suggesting that close proximity between pDCs and B cells may be required. Importantly, Rag2^−/−^ pDCs were also able to promote antibody class switching and plasma cell formation (Fig. 6*A–C*), suggesting that the lack of Rag2 may not block the function of pDCs in supporting B cells. We next asked whether IL-6 mediated the effect of Rag2^−/−^ pDCs on antibody class switching from IgM to IgG. We found that both Rag2^+/+^ and Rag2^−/−^ pDCs naturally secreted IL-6 (Fig. 6*D*) and that a blocking antibody against IL-6 was able to abolish the effects of both types of pDCs on antibody class switching (Fig. 6*E–F*), suggesting that although Rag2^−/−^ pDCs are defective at producing IFNα, they are able to support antibody class switching through producing IL-6.

Altogether, these results suggest that in pDCs, Rag2 specifically regulates CpG-mediated induction of IFNα, whereas other functions of pDCs may be intact in the absence of Rag2.

### Dysregulation of IRF8 in Rag2^−/−^ pDCs

**Figure 7:**
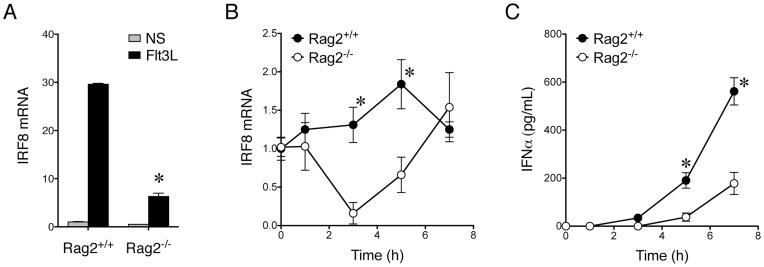
Dysregulation of IRF8 expression in Rag2^−/−^ pDCs. *A.* Induction of IRF8 mRNA expression in pDCs with Flt3 ligand. PDCA-1^+^ pDCs were sorted and either untreated (NS) or treated with Flt3 ligand (Flt3L) for 5 days. Total RNA was isolated and RT-qPCR was performed. IRF8 mRNA levels were normalized to L32 and the expression level in Rag2^+/+^ pDC without stimulation was defined as 1. The difference in Flt3L-induced IRF8 expression between Rag2^+/+^ and Rag2^−/−^ pDCs was significant (n  = 3, *p*<.05). *B.* Time course of IRF8 mRNA expression in pDCs upon CpG stimulation. Rag2^+/+^ and Rag2^−/−^ PDCA-1^+^ pDCs were sorted and treated with ODN1585 (CpG) for 1, 3, 5, or 7 h. Total RNA was isolated and RT-qPCR was performed. IRF8 mRNA levels were normalized to L32 and the expression level in Rag2^+/+^ pDC without stimulation was defined as 1. The asterisks represent significant differences (n  = 3, *p*<.05) between IRF8 levels in Rag2^+/+^ versus Rag2^−/−^ pDCs at 3 and 5 h. *C.* Time course of IFNα production in pDCs upon CpG stimulation. Rag2^+/+^ and Rag2^−/−^ PDCA-1^+^ pDCs were sorted and treated with ODN1585 (CpG) for 1, 3, 5, or 7 h. IFNα production were measured by using ELISA. The asterisks represent significant differences (n  = 3, *p*<.05) between IFNα levels in Rag2^+/+^ versus Rag2^−/−^ pDCs at 5 and 7 h.

To elucidate the mechanism by which Rag2 regulates CpG-mediated induction of IFNα in pDCs, we examined the expression level of IRF8, a transcription factor important for both pDC development [47] and IFNα production [48]. We found that during the expansion of pDCs by Flt3 ligand, IRF8 mRNA was dramatically upregulated in bone marrow-derived wildtype and Rag1^−/−^ pDCs (Fig. 7*A* and Fig. S4). Rag2^−/−^ pDCs, however, expressed IRF8 at a significantly lower level upon the stimulation (Fig. 7*A*, *p*<.05). This suggests that pDCs may be developmentally impaired in the absence of Rag2. In addition, we examined the change of IRF8 mRNA levels over time after CpG stimulation. We found that IRF8 mRNA was slightly upregulated in Rag2^+/+^ but not Rag2^−/−^ pDCs (Fig. 7*B*). The levels of IRF8 mRNA was significantly lower in Rag2^−/−^ pDCs at 3 h and 5 h after CpG stimulation (Fig. 7*B*, *p*<.05), which might have led to a significant difference in the magnitude of IFNα production observed between Rag2^+/+^ and Rag2^−/−^ pDCs (Fig. 7*C*, *p*<.05). These results suggest that IRF8 expression is dysregulated in Rag2^−/−^ pDCs, which might be one reason why CpG-mediated IFNα production is defective in pDCs lacking Rag2.

## Discussion

Using Rag2^−/−^ mice, we have found that pDCs as main producers of IFNα require Rag2 for normal development. This is a novel function for Rag2, whose classical role is to initiate B and T cell development. Our results showed that, although the numbers of pDCs were similar between Rag2^+/+^ and Rag2^−/−^ mice, Rag2^−/−^ pDCs produced a significantly lower level of IFNα in vitro and in vivo in response to CpG, a ligand for TLR9. Flt3 ligand, a known stimulator of pDC proliferation, could expand Rag2^−/−^ pDCs in vitro; however, the expanded cells expressed a high level of Ly6C. These Ly6C^hi^ pDCs were defective at producing IFNα in response to CpG. In contrast, Rag2^−/−^ pDCs retained the function to induce CCR7, CD40, and CD86 with CpG. They were also able to promote antibody class switching and plasma cell formation through producing IL-6. These results suggest Rag2 as a novel regulator of IFNα production in pDCs.

During B and T cell development, Rag2 couples with Rag1 to regulate V(D)J recombination. Although Rag2 is generally thought to function by pairing with Rag1, recent evidence has shown that only Rag2 is essential for maintaining genomic stability since knockout of this protein, but not knockout of Rag1, on p53^−/−^ background leads to rapid formation of thymic lymphomas [49]. This suggests that Rag2 may have unique functions that are not shared by Rag1. We show here that Rag2^−/−^ pDCs expressed a high level of Ly6C but Rag1^−/−^ pDCs expressed a similar amount of Ly6C as wildtype pDCs (Fig. 3*C* and Fig. S2). In addition, Rag2^−/−^ bone marrow cells had a defect in CpG-mediated IFNα induction but Rag1^−/−^ bone marrow cells did not (Fig. 4*A*, left panel and Fig. S3). Moreover, while IRF8 mRNA was dramatically upregulated by Flt3 ligand in both wildtype and Rag1^−/−^ pDCs, the increase of IRF8 was much lower in pDCs lacking Rag2 (Fig. 7*A* and Fig. S4). The lack of such defects in Rag1^−/−^ pDCs suggests that maturing pDCs may not need the presence of lymphocytes to become fully competent. These observations also suggest that Rag2 may be able to function separately from Rag1.

PDCs as main producer of IFNα function as fine tuners of immune responses. An inadequate amount of IFNα due to the lack of functional pDCs can lead to uncontrolled viral infection and/or cancer [1]. Too much of IFNα, on the other hand, can cause autoimmune disorders [50]. Therefore, the function of pDCs to produce IFNα must be tightly controlled. This function of pDCs parallels the production of IFNα by conventional dendritic cells, which is mediated by retinoic acid-inducible gene I (RIG-I)-dependent signaling [51]. The production of IFNα by pDCs, however, is triggered through TLR7 and TLR9 ligation [34]. The TLR-mediated signaling cascade involves myeloid differentiation primary response gene 88 (MyD88)/tumor necrosis factor receptor-associated factor 6 (TRAF6)/interleukin 1 receptor-associated kinase 1 (IRAK1) pathway and subsequent phosphorylation of IRF7. It has also been shown that TLR-mediated induction of IFNα in pDCs requires phosphatidylinositol-3-OH kinase (PI3K)/Akt/mammalian target of rapamycin (mTOR) signaling pathway [52] and nuclear factor-κB (NF-κB) and p38 mitogen-activated protein kinase (MAPK) signaling pathways [53]. Recently, an antiviral protein that is induced by interferon signaling has been shown to promote TLR-mediated IFNα production in pDCs, suggesting a positive feedback loop that controls the primary function of pDCs to produce IFNα [54]. We show here that Rag2 also regulates IFNα production in pDCs. In addition, we have found that Rag2 regulates IFNα production without affecting other functions of pDCs–which include the expression of surface functional markers and the support of antibody class switching–suggesting that Rag2 may specifically regulate the signaling events leading to IFNα induction.

We focused on IRF8 to elucidate the role of Rag2 in IFNα induction because this transcription factor is involved in not only IFNα induction, but also pDC development. IFNα is induced in two phases upon viral infection [48]. The second amplifying phase, which produces more IFNα than the first phase, requires IRF8. IRF8 is also required for the pDC maturation [47], a process that is facilitated by Flt3 ligand and inhibited by granulocyte macrophage colony-stimulating factor (GM-CSF) [55]. In order for GM-CSF to block pDC formation, it uses signal transducers and activators of transcription-5 (STAT5) to directly suppress the expression of IRF8 [56]. In this study, we determined IRF8 expression in Rag2^−/−^ versus Rag2^+/+^ pDCs upon Flt3 ligand or CpG stimulation, and found that IRF8 mRNA was dysregulated in pDCs lacking Rag2 (Fig. 7). How Rag2 is involved in the dysregulation of IRF8 and whether additional pDC-specific transcription factor such as E2-2 [57], [58] are altered in Rag2^−/−^ pDCs require further investigation. In addition, it has been recently reported that CCR9**^−^**MHC-II^low^ pDCs produce more IFNα than CCR9^+^MHC-II^+^ pDCs [22]. We did not observe any difference in MHC-II expression between Rag2^+/+^ and Rag^−/−^ pDCs (data not shown), but we have not analyzed CCR9 and will be interested in investigating whether Rag2 regulates CCR9 expression in future studies. We are also interested in knowing whether pDCs isolated from Rag2^−/−^ mice would differ in the expression of Rag1 and other B-lineage associated genes. Furthermore, we will try other TLR stimulations and compare the response to those triggered by TLR9 ligands. TLR7/8 and TLR9 ligands have been shown to trigger different inflammatory responses [59] and it will be also interesting to find out why CpG-mediated, but not TLR7/8 ligand-mediated production of IFNα, is affected by Rag2 expression.

Overall, our results have shown that Rag2, an essential protein for B and T cell development, may be also important for the development of the primary function of pDCs as professional interferon-producing cells. This suggests a common early developmental path for B/T cells and pDCs. This also suggests Rag2 as a novel therapeutic target for the treatment of excessive interferons such as those in autoimmune disorders.

## Supporting Information

Figure S1 **Numbers of pDCs in the mouse bone marrow.** Bone marrow cells from wildtype (WT), Rag1^−/−^, and Rag2^−/−^ mice (n  = 4 in each group) were isolated, counted, and analyzed by using flow cytometry. The percentages of PDCA-1^+^ pDCs were determined and used to calculate the numbers of pDCs.
(TIF)

Figure S2 **Ly6C expression during Flt3 ligand-mediated expansion of Rag1^−/−^ CD11b^−^CD11c^+^ cells.** Rag1^−/−^ total bone marrow cells were either untreated or treated with Flt3 ligand for 5 days. CD11b**^−^**CD11c^+^ cells were gated and analyzed for Ly6C expression. Representative plots of 3 independent experiments are shown.
(TIF)

Figure S3 **Rag1^−/−^ pDCs normal at IFNα production upon CpG stimuation.** Bone marrow cells were isolated from wildtype (WT) and Rag1^−/−^ mice (n  = 4 in each group). Cells were either not stimulated (NS) or stimulated with ODN1585 (CpG) in vitro for 5 days. Production of IFNα in the medium was measured by using ELISA.
(TIF)

Figure S4 **IRF8 mRNA expression in pDCs upon Flt3 ligand stimulation.** Wildtype (WT) or Rag1**^−^** ^/−^ PDCA-1^+^ pDCs (n  = 3 in each group) were sorted and either untreated (NS) or treated with Flt3 ligand (Flt3L) for 5 days. Total RNA was isolated and RT-qPCR was performed. IRF8 mRNA levels were normalized to L32 and the expression level in WT pDC without stimulation was defined as 1.
(TIF)
