# Supplementary figures and images for: Recombination Activating Gene-2 Regulates CpG-Mediated Interferon-α Production in Mouse Bone Marrow-Derived Plasmacytoid Dendritic Cells
Source: PLoS One. 2012 Oct 24;7(10):e47952. doi: 10.1371/journal.pone.0047952 (PMC3480463; doi:10.1371/journal.pone.0047952)

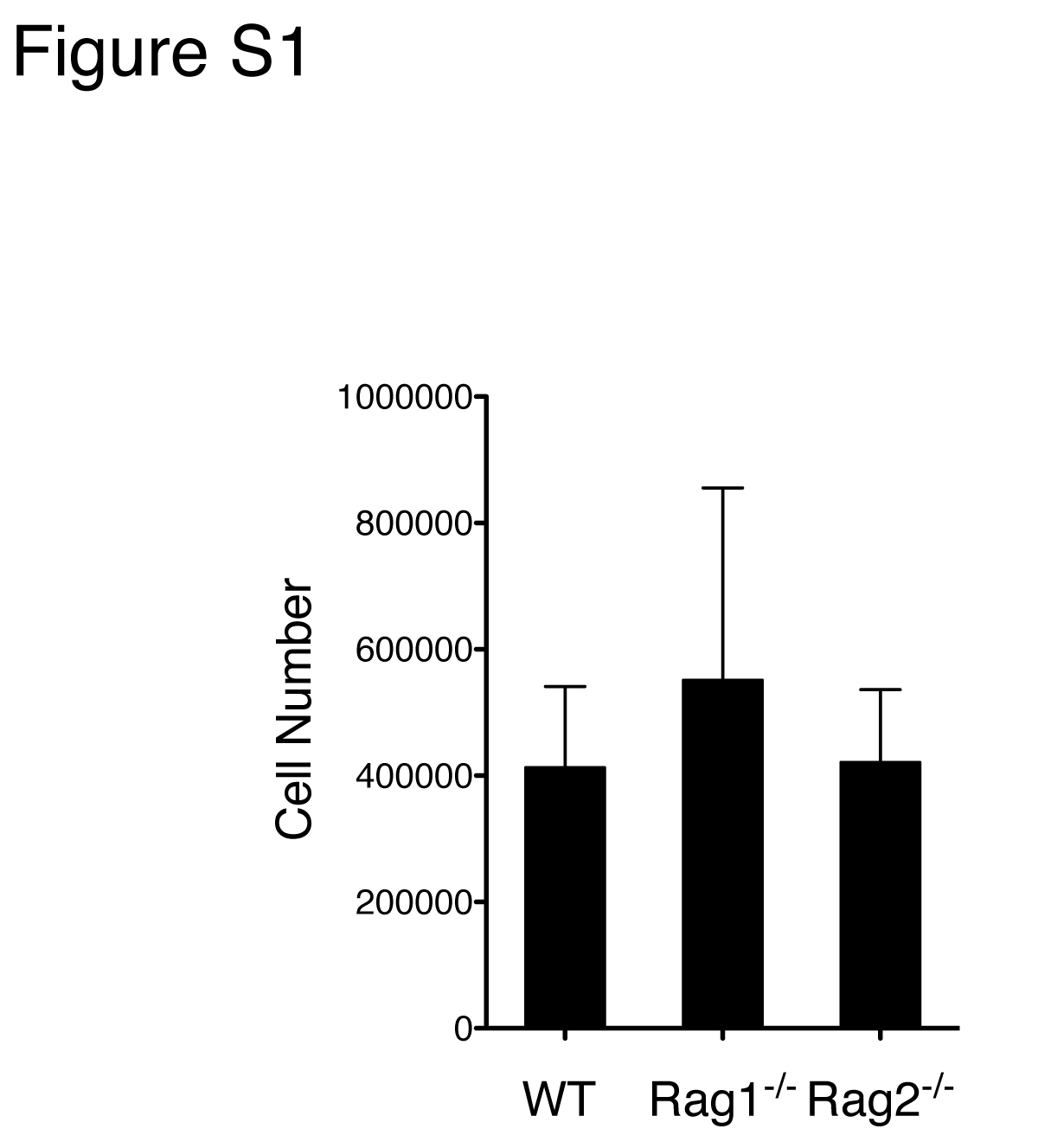

Supplement: Figure S1 — Numbers of pDCs in the mouse bone marrow. Bone marrow cells from wildtype (WT), Rag1−/−, and Rag2−/− mice (n = 4 in each group) were isolated, counted, and analyzed by using flow cytometry. The percentages of PDCA-1+ pDCs were determined and used to calculate the numbers of pDCs. (TIF) [file pone.0047952.s001.tif]

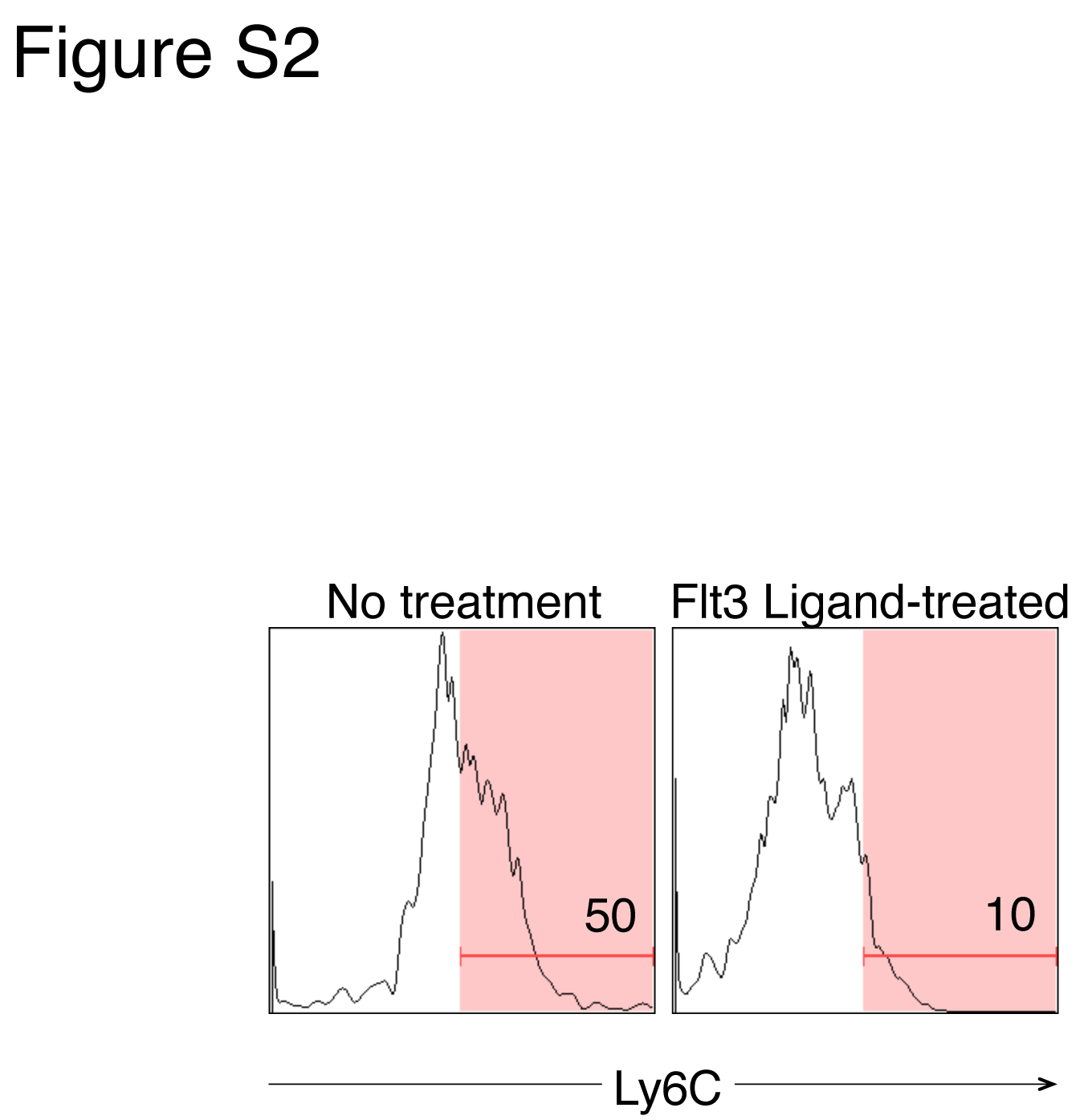

Supplement: Figure S2 — Ly6C expression during Flt3 ligand-mediated expansion of Rag1−/− CD11b−CD11c+ cells. Rag1−/− total bone marrow cells were either untreated or treated with Flt3 ligand for 5 days. CD11b−CD11c+ cells were gated and analyzed for Ly6C expression. Representative plots of 3 independent experiments are shown. (TIF) [file pone.0047952.s002.tif]

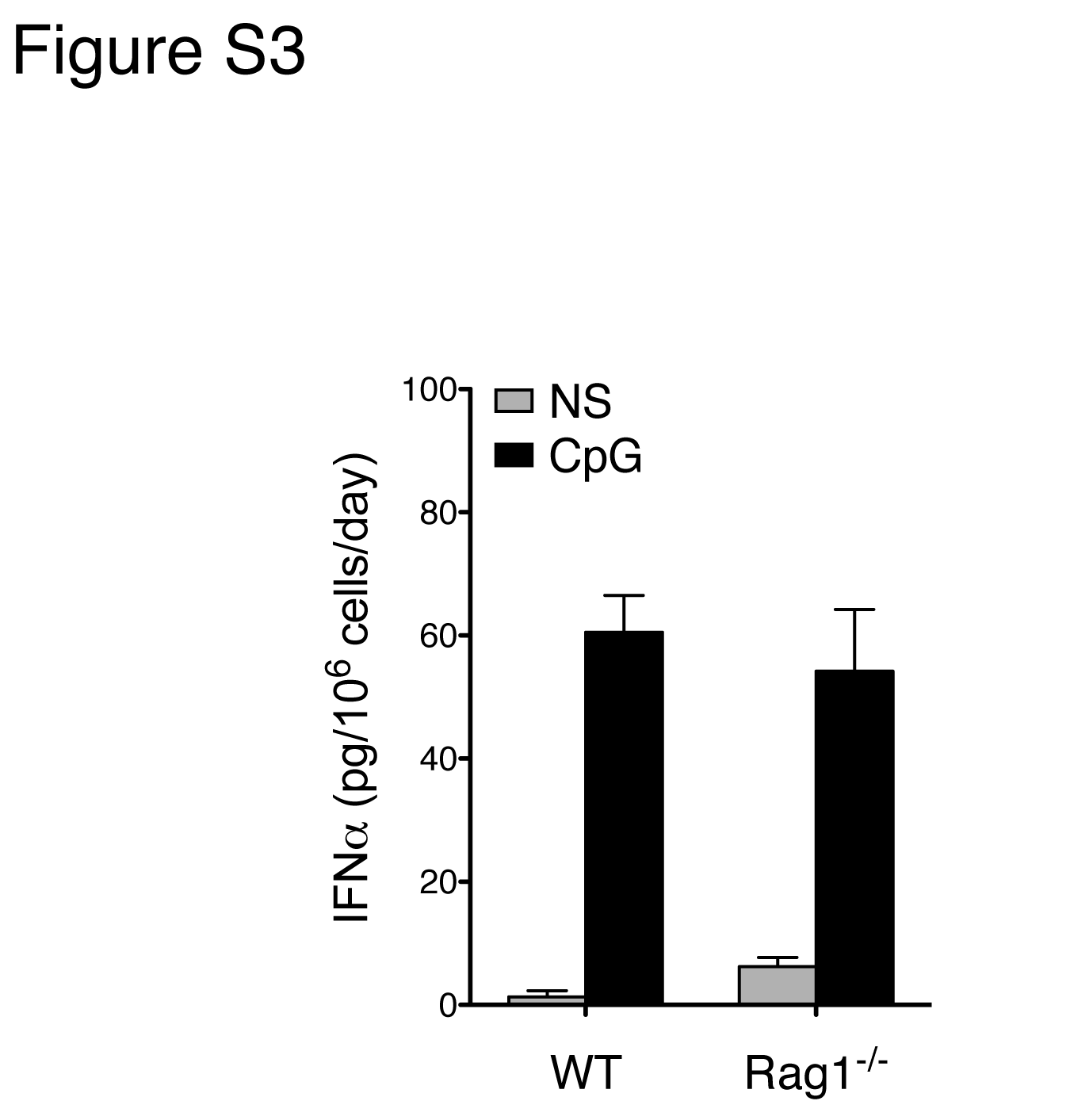

Supplement: Figure S3 — Rag1−/− pDCs normal at IFNα production upon CpG stimuation. Bone marrow cells were isolated from wildtype (WT) and Rag1−/− mice (n = 4 in each group). Cells were either not stimulated (NS) or stimulated with ODN1585 (CpG) in vitro for 5 days. Production of IFNα in the medium was measured by using ELISA. (TIF) [file pone.0047952.s003.tif]

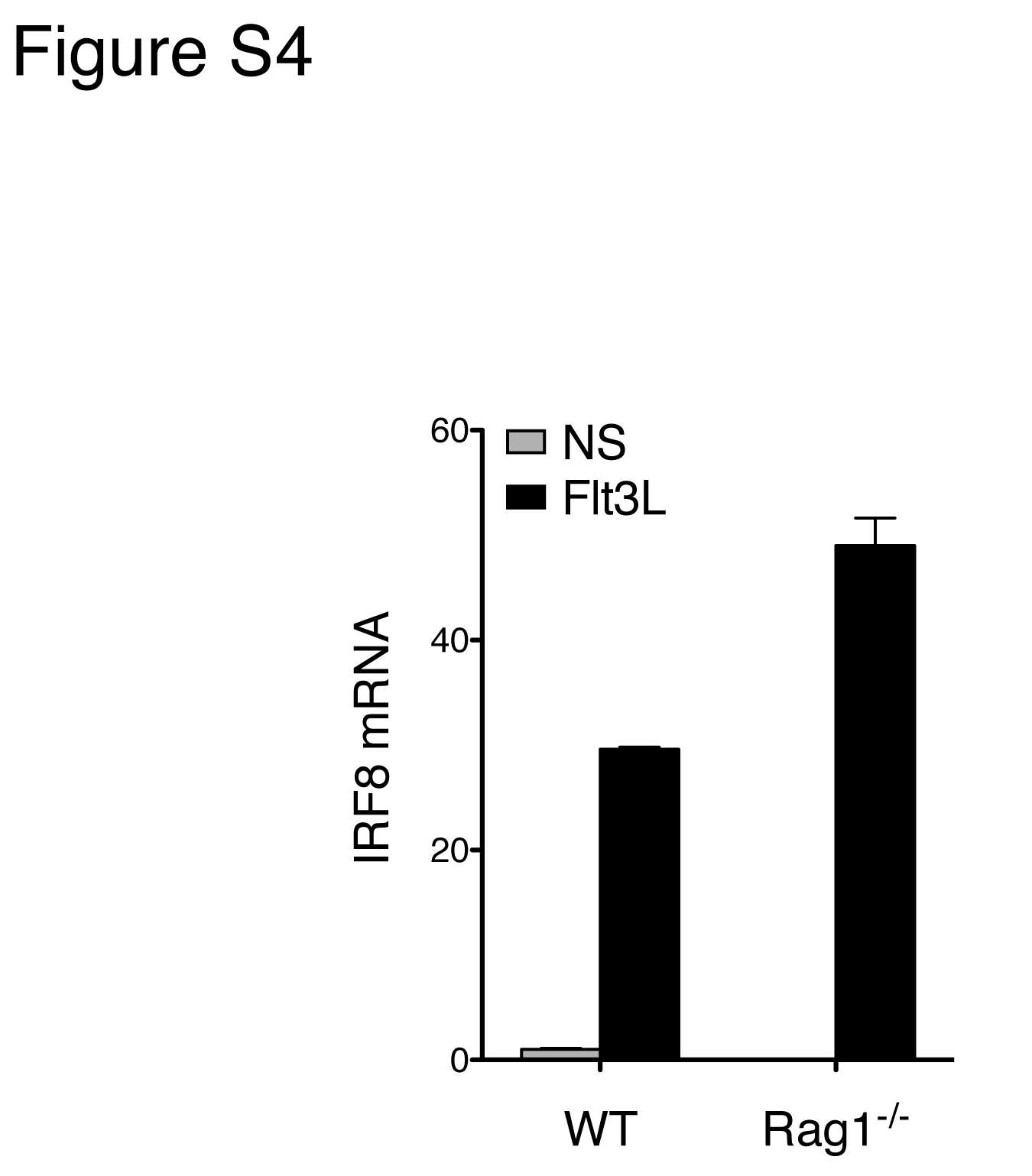

Supplement: Figure S4 — IRF8 mRNA expression in pDCs upon Flt3 ligand stimulation. Wildtype (WT) or Rag1− /− PDCA-1+ pDCs (n = 3 in each group) were sorted and either untreated (NS) or treated with Flt3 ligand (Flt3L) for 5 days. Total RNA was isolated and RT-qPCR was performed. IRF8 mRNA levels were normalized to L32 and the expression level in WT pDC without stimulation was defined as 1. (TIF) [file pone.0047952.s004.tif]
